# The Assessment and Efficiency of Cefixime in Upper Respiratory Tract Infections: Insights and Perspectives

**DOI:** 10.7759/cureus.64539

**Published:** 2024-07-14

**Authors:** Mohammad K Shafi, Azher A Shah, Muhammad A Khan, Sarwat Faisal, Sarmad Iqbal

**Affiliations:** 1 Community Medicine, Dow University of Health Sciences, Karachi, PAK; 2 Pediatric Medicine, University of Child Health Sciences, Lahore, PAK; 3 Pediatric Medicine, Hayatabad Medical Complex, Peshawar, PAK; 4 Medicine, College of Family Medicine, Karachi, PAK; 5 Pharmacy Practice, University of Karachi, Karachi, PAK

**Keywords:** clinical practice, respiratory tract infections, insights, pediatrics, cefixime

## Abstract

Upper respiratory tract infections (URTIs) are common in patients of the pediatric age group and often lead to significant morbidity and mortality. Antibiotics such as cefixime have contributed to the management of URTIs, particularly when bacterial etiology is suspected. Several studies have evaluated the effectiveness of cefixime in pediatric URTIs, showing promising results in alleviating symptoms and reducing the duration of illness.

Cefixime, a third-generation cephalosporin, exhibits broad-spectrum activity against common pathogens implicated in URTIs, including *Streptococcus pneumoniae*, *Haemophilus influenzae*, and *Moraxella catarrhalis*, which are resistant to hydrolysis by several β-lactamases. Due to its unique three-hour elimination half-life, cefixime allows for twice-daily or, in most cases, once-daily dosage. As a third-generation cephalosporin, cefixime effectively targets the common bacterial pathogens associated with these infections. Its notable efficacy is coupled with a favorable safety profile, making it a preferred choice for pediatricians and family physicians.

The safety profiles of cefixime in children have been extensively studied with generally favorable outcomes. Adverse events are typically mild and infrequent, with gastrointestinal disturbances being most commonly reported. Notably, cefixime has a low propensity to induce bacterial resistance, making it a valuable option in the era of increasing antibiotic resistance. Cefixime may serve as a substitute for penicillin and first-generation cephalosporins in cases of acute upper and lower respiratory tract infections, acute otitis media, and acute uncomplicated urinary tract infections. This review aimed to provide a comprehensive outline of the use of cefixime in the treatment of URTIs in the pediatric population, focusing on its efficacy, safety, and overall clinical applications.

## Introduction and background

The beta-lactam class of antibacterial agents includes cephalosporins and penicillin. Cephalosporins were first recognized in 1945 by the Italian chemist Giuseppe Brotzu (1895-1976), who extracted a combination of chemicals from the mold *Acremonium*, formerly known as *Cephalosporium *[1]. Later, in 1955, as a minor part of the combination of antibiotics generated by *Acremonium*, the British scientists Edward Abraham (1913-1999) and Guy Newton (1919-1969) identified, refined, and reported the chemical composition of cephalosporin C [2]. Prior to the advent of semisynthetic cephalosporins in 1960, cephalosporin C had very little antibacterial activity and was synthesized in very small amounts [3].

These antibiotics have a six-member dihydrothiazine ring attached to the beta-lactam component of their structure. Their antibacterial action is mostly dependent on the substituting elements at positions C3, C4, and C7. Additionally, the carboxyl group at C4 must not change, and the hydrophilic and hydrophobic characteristics of these compounds are largely dependent on the acylamido side chain at C7 (7-aminocephalosporanic nucleus) [4].

The synthesis of cephalosporins involves structural change in a lab setting. These antibiotic agents are divided into five generations, the first through the fifth, according to the chronology of their discovery and their antimicrobial characteristics [4]. The difference pertains to the molecular structure and has significant therapeutic implications.

The antimicrobial effects of cephalosporins rise against gram-negative bacilli but decline in opposition to gram-positive organisms as they progress from the first to the third generation. It is noteworthy that from the first to the fifth generation, there is a rise in resistance to beta-lactamases [5].

First-generation cephalosporins, like cefadroxil, cefazolin, and cephalexin, are only effective against gram-positive bacteria with respect to their antimicrobial activity. Additionally, despite the fact that there is reduced activity against gram-positive bacteria, second-generation antibiotics (such as cefotetan, cefaclor, cefamandole, and loracarbef) have greater effectiveness against gram-negative and some anaerobes [1].

The most often prescribed class of cephalosporins is the third generation. These semisynthetic analogs of cephalosporins have distinct chemical modifications on the C7 acylamido chain. There are several more medications in this family as well, such as cefixime, ceftriaxone, cefdinir, cefditoren, cefpodoxime, ceftazidime, cefoperazone, ceftizoxime, and ceftibuten. They are antimicrobial drugs with an extensive range of action that can combat both gram-positive and gram-negative bacteria. However, they are more effective against pathogens resistant to the first and second generations of cephalosporins, as well as gram-negative bacteria [6].

Additionally, it appears that these medicines have reduced effectiveness against a variety of gram-positive bacteria, including species of *Staphylococcus *and *Streptococcus*. Alternatively, they exhibit some degree of activity against gram-positive pathogens, albeit not to the same extent as the first-generation cephalosporins.

Remarkably, third-generation cephalosporins, particularly those generated by *Klebsiella*, *Haemophilus influenzae*, and *Escherichia coli*, show greater efficacy against beta-lactamases than do first- or second-generation cephalosporins. The rest of the class is inactive in opposition to *Pseudomonas aeruginosa*, while cefoperazone and ceftazidime are active. In patients with healthcare-associated and community-acquired spontaneous bacterial peritonitis (SBP) without hepatocellular cancer, third-generation cephalosporins seem to provide appropriate empirical therapy in spite of the growing number of gram-positive organisms in SBP [6].

## Review

Dosage and administration

The administration routes for third-generation cephalosporins are intramuscular, intravenous, and oral. Effective oral medications for outpatient settings include cefixime, ceftibuten, cefdinir, cefpodoxime, and cefditoren. All oral medications, except ceftibuten and cefdinir, are esters that are degraded by esterases in the gastrointestinal system for absorption [7]. These medications have minimal risk for toxicity, proven therapeutic plasma concentrations, and great oral bioavailability.

Adults typically receive 400 mg per day in one dose or in two equally distributed doses. For simple urinary tract infections, a lower dosage of 200 mg per day has been recommended. In children, the most common dosage for treating acute tonsillitis, acute pharyngitis, and acute otitis media of cefixime is 8 mg/kg/day, once daily or in two divided doses. Patients with significant renal impairment (creatinine clearance <20 ml/min) should receive half the recommended daily dose of cefixime [8].

Pharmacokinetic properties

Cefixime's highest plasma concentrations following oral administration typically occur three to four hours after a single 200 mg dose and range from 2.0-2.6 mg/L (mean).

Food has no effect on the pharmacokinetics of cefixime other than delaying the time to reach its highest concentrations in the plasma. After 200 mg administered two times a day or 400 mg administered once a day for 15 days, there is no sign of drug deposits. Cefixime dose at 8 mg/kg/day in children had pharmacokinetics that matched those of adults taking 400 mg. Cefixime's estimated absolute bioavailability was 48% for 200 mg capsules, 52% for an oral solution form, and 40% for 400 mg capsules [9].

Following a single intravenous dose, the apparent volume of distribution was 6.7L. The distribution's volume was roughly 17 liters in a normal state. The palatine tonsil and maxillary sinus mucosa showed drug concentrations of 0.2 to 0.8 mg/kg and 0.5 to 1.05 mg/L, respectively, three to five hours after single 100 mg doses. Sputum showed drug concentrations of 0.04 to 0.06 mg/L six to eight hours after a 100 mg dose. After taking 100 mg of cefixime once or more, middle ear secretions showed concentrations ranging from 0.09 to 1.46 mg/L. Bile contains high quantities. After about 0.5 to five hours following a 100 mg dose, the concentrations of cefixime in umbilical cord serum were around 15 to 30% of those in maternal plasma. In individuals in good health, cefixime is roughly 70% bound to proteins [9].

During the course of a 24-hour period, the urine retains an average of 12 to 20% of a 200 mg dose, with no physiologically active metabolites seen in either plasma or urine. Following intravenous treatment, the total systemic clearance was approximately 4.4 L/h (73 ml/min), of which approximately 40% was due to renal clearance.

Following a 200 or 400 mg dosage, the oral clearance was 9.7 and 11.4 L/h (150 and 190 ml/min), respectively. The elimination half-life during the end stage is typically around three hours for healthy individuals. However, this is only significantly longer in patients with impaired renal function whose creatinine clearance is less than 20 ml/min, which means that dosage adjustments would be required [9]

Safety profile

Previous studies reported that most clinical side effects in cefixime-treated patients are low to moderate in severity and usually are of a temporary nature. The most often reported side effects have been loose stools and changes in stool frequency (as contrasted with diarrhea), which occurred in 13.8 and 13.5% of patients, respectively.

In adults, diarrhea tends to occur more frequently (15.3%) after once-daily treatment as opposed to twice-daily (10.3%); however, in children, this pattern was not evident. In around 63% of cases, diarrhea was noticeable within four days of starting treatment, which is not the usual pattern associated with alterations in gut flora. Comparative studies carried out and demonstrated that diarrhea occurs frequently with cefixime in comparison to amoxicillin, whereas other gastrointestinal problems happened with the same frequency with both medications [10].

The use of third-generation cephalosporins has a similar risk of super-infection to other classes and subclasses of antibiotics. Additionally, cases of third-generation cephalosporin-induced pseudomembranous colitis due to *Clostridium difficile* have been reported [11]. There have been cases of hypersensitivity reactions; however, severe allergic reactions are rare. In the general community, cephalosporin allergies affect 1-3 percent of people [12]. Cephalosporins function as hapten and may trigger antibody responses in immune-mediated hemolytic anemia and thrombocytopenia. In patients who are hypersensitive to cephalosporins, antihistamines, corticosteroids, epinephrine, or vasopressors are used to prevent anaphylactic reactions [13].

Seizures and responses similar to those of disulfiram are two more uncommon side effects of certain third-generation cephalosporins. Besides the common epileptogenic activity, cephalosporin-induced neurotoxicity might manifest clinically as myoclonus, asterixis, or encephalopathy, amongst other symptoms. Though the pathogenic mechanism is unclear, it is most likely connected to GABA's competitive antagonistic effects. There is a very small risk of ceftriaxone-induced encephalopathy, which can include diminished memory, behavioral issues, weakness in the arms and legs, or tingling [14]. From a clinical perspective, the symptoms appear one to seven days into antibiotic therapy, and they typically resolve within two to seven days after the drug is discontinued. However, no nephrotoxicity associated with third-generation cephalosporins has been reported. Biliary pseudolithiasis is the end result of ceftriaxone's binding of calcium in the bile and the subsequent formation of stones [15].

Since cefdinir and ceftriaxone can also induce Steven-Johnson syndrome, cephalosporins fall into the high-risk category of drugs that can cause this condition [16]. One of the main factors contributing to severe cutaneous adverse events and perioperative anaphylaxis is cephalosporin usage [12]. In addition, a fungal infection, diarrhea, rashes or itchiness, injection site responses, nausea, vomiting, and digestive problems are more frequent but less serious side effects.

While the safety profile of cefixime in the pediatric population is previously established, rare reports of Steven Johnson syndrome (SJS) and acute generalized exanthematous pustulosis (AGEP) have been recently reported in the pediatric population in India and Bangladesh [17, 18].

Pathogens causing upper respiratory tract infections

Multiple different bacteria, including mycobacteria and viruses, can cause upper respiratory tract infections (URTIs). In order to achieve the optimum therapeutic therapy, it is imperative to identify the pathogens in individual cases as soon as possible [19]. *Moraxella catarrhalis*, *Haemophilus influenzae*, *Streptococcus pyogenes*, and *Mycoplasma pneumoniae* are the most frequent bacteria that cause URTIs. Clinical manifestations of these infections include tonsillitis, pharyngitis, epiglottitis, sinusitis, rhinitis, bronchitis, and nasopharyngitis [20]. Although only 10% of cases are linked to bacteria, the majority of infections are typically viral because bacterial super-infections are frequently found and superimposed on earlier viral illnesses, making it challenging to determine the specific percentage of bacterial infection; hence, where superadded infections are suspected, antibiotics are used to treat the majority of patients [21, 22].

The use of antibiotics may be justified by the theory that viral infections that compromise the integrity of the airway epithelium allow bacterial pathogens to enter the body, induce inflammation, raise the vascular endothelium's permeation, and frequently result in bronchial hyperreactivity. There have been reports of a variety of URTI signs and symptoms, such as nasal congestion or runny nose, coughing, sneezing, throat irritation, nausea, fever, loss of appetite, and watery eyes. Microorganisms responsible for URTIs can spread through aerosol, droplets, and direct hand-to-hand contact with contaminated fluids. Compared to adults, children are more vulnerable to URTIs, which may be brought on by children's intimate person-to-person contact and lack of antibodies to the various viruses and bacteria that lead to URTIs [23-25].

The unusual bacterium *Chlamydia pneumoniae* is a major contributor to URTIs. This intracellular bacterium is gram-negative and is reliant on ATP accumulation from the host cell to form aggregates in the cytoplasm of infected cells. The two forms of *Chlamydia pneumoniae*, the reticulate body (RB), which is intracellularly metabolically active, and the elementary body (EB), which is responsible for propagating the infection, have a distinct developmental cycle. In addition to this acute infection-replicating cycle, *Chlamydia pneumoniae* can also live in a non-replicating condition [26, 27].

Microbial interactions during upper respiratory tract infections

The bacteria that are most commonly associated with community-acquired respiratory tract infections are *Streptococcus pneumoniae*, *Haemophilus influenzae*, β-hemolytic streptococci (which are often members of Lancefield group A), and *Moraxella catarrhalis*. These species may, therefore, be responsible for conditions such as acute exacerbations of chronic bronchitis, pharyngitis, sinusitis, otitis media, and acute bronchitis. In spite of the generation of β-lactamase, cefixime has high action against these pathogens. Pneumococci resistant to cefixime are also resistant to penicillin; however, these strains are still uncommon in most regions of the world. Cefixime's spectrum of action is, therefore, appropriate for infections affecting this particular physiological system [28].

Role of first-line antibiotics in upper respiratory tract infection

Antibiotic use in cases of acute rhinosinusitis is still debatable. Sinusitis is caused by a viral upper respiratory infection and may not be treated with antibiotics. Typically, a bacterial infection coexists in just one to two out of every 100 otherwise healthy individuals experiencing nasal symptoms [29]. Differentiating patients who will heal on their own from those who will need antibiotic medication can often be challenging. Many times, there is no proof of any bacterial, fungal, or viral cause for the illness; in fact, the illness may just be an expression of an inflammatory process. For these people, the benefits of antibiotics would be negligible or nonexistent. Thus, it is essential to make an effort to identify the patients who will benefit from antibiotic therapy in order to prevent the excessive use of antibiotics, which may add to the emergence of bacterial resistance.

Amoxicillin

High-dosage amoxicillin (90 mg/kg/day) ought to be the primary choice for treating sinusitis in view of its effectiveness against sinus infections. The incorporation of clavulanic acid into amoxicillin offers a benefit over amoxicillin alone since the percentage of cases brought on by *H. influenza* is probably growing, and this organism is producing more B-lactamases at a faster speed. For *S. pneumoniae* that is not susceptible to penicillin, using the amoxicillin component at a dosage of 90 mg/kg/day is considered to be more effective [30].

Oral cephalosporins

The most often prescribed class of cephalosporins is the third generation. There are several more medications in this group as well, such as ceftriaxone, cefdinir, cefixime, cefditoren, cefpodoxime, ceftazidime, cefoperazone, ceftizoxime, and ceftibuten. They are antimicrobial drugs with a broad spectrum of action that can be effective against both gram-positive and gram-negative bacteria. Notably, third-generation cephalosporins showed greater efficiency against beta-lactamases, as compared to first- or second-generation [31]. Oral cephalosporins are ineffective against *S. pneumonia*,* *which is resistant to penicillin [32, 33]. In place of high-dose amoxicillin-clavulanate, injectable third-generation cephalosporins, cefotaxime, and ceftriaxone are the recommended second-line empirical therapy for hospitalized children. They are efficacious against all strains of *S. pneumoniae*, including penicillin-resistant strains [34, 35]. The most effective oral cephalosporins against beta-lactamase-positive and negative *H. influenzae* and *M. catarrhalis* are cefixime, cefuroxime, and cefdinir; cefaclor and cefprozil are less effective [32, 36].

Cefixime is a group of the third-generation of cephalosporin antibiotics. It damages the cell wall of bacteria by binding to proteins that bind penicillin and preventing the formation of peptidoglycan. The broad spectrum action of third-generation cephalosporins against all gram-negative and positive infections as well as atypical species, including *Mycoplasma *and *Chlamydia*,* *makes them widely applicable [37]. It is established that the effectiveness of oral cephalosporins of the second and third generations against *S. pneumoniae* and *H. influenzae* varies significantly. Therefore, as monotherapy, these medicines are no longer suitable for the initial empirical treatment of URTIs in children. In areas with high isolation rates of penicillin-resistant *S. pneumonia*, it is advisable to administer a third-generation cephalosporin orally (such as cefpodoxime or cefixime) in combination with clindamycin.

Cefixime use in children

Often, cefixime works well as an antibiotic, but there are many inexpensive and equally effective options. Using cefixime in children should adhere to the following recommendations:

Penicillin V is still the recommended treatment for streptococcal pharyngitis. Research has shown that penicillin is the only medication that effectively prevents rheumatic fever. However, erythromycin and cephalosporins are good substitutes for penicillins. For the treatment of streptococcal sore throat, cefixime and other first- and second-generation cephalosporins work effectively [8].

Concerning urinary tract infection, cefixime gives less benefit in comparison with inexpensive medications like amoxicillin, trimethoprim-sulfamethoxazole, and nitrofurantoin. Nevertheless, urinary infections that are sensitive to cefixime but resistant to those antibiotics can be treated with cefixime. In those cases where quinolones are not recommended, cefixime is an effective therapy for gastroenteritis in young children, which is caused by strains of *Shigella *and *Salmonella*, and is resistant to commonly used antibiotics such as trimethoprim-sulfamethoxazole and amoxicillin. It is unknown, nonetheless, if the growing usage of cefixime may lead to the eventual development of resistance in these infections.

Based on preliminary data, transitioning from early parenteral antibiotic therapy to oral cefixime may be a useful substitute in mitigating the risk of severe Gram-negative infections in cancer patients recovering from febrile neutropenia [8, 38], though controlled trials are still needed in this regard.

Effectiveness, safety, and clinical relevance of cefixime for pediatric respiratory tract infections

The purpose of this open-label, randomized study was to evaluate the safety and effectiveness of cefixime against ciprofloxacin in the empirical management of community-acquired pneumonia in adult patients from Nigeria. For 14 days, they received treatment with either 400 mg of cefixime or 500 mg of ciprofloxacin twice a day. Cefixime proved to be more effective than ciprofloxacin in treating adult patients with community-acquired pneumonia. Nonetheless, there were no reports or records of adverse events indicating that all patients tolerated the two medications well [39].

Similarly, there was a prospective trial to evaluate cefixime's clinical effectiveness, bacteriological eradication rates, and tolerability among children with community-acquired upper RTI (URTI), lower RTI (LRTI), and uncomplicated UTIs. Cefixime is an excellent option for an effective clinical response for treating community-acquired infections like acute otitis media (AOM), LRTI, and UTI brought on by susceptible pathogens. The findings of this study indicate that cefixime is effective in treating individuals with community-acquired infections, including UTI, LRTI, and acute otitis media. Cefixime should not be used as a treatment for acute infections when *Staphylococcus aureus* is suspected to be the pathogen; instead, another antibiotic should be used, according to vulnerability at the antibiogram. Cefixime exhibited good tolerability, negating the need to stop treatment. They found that patients' and parents' adherence to the cefixime treatment protocol for AOM, LRTI, and UTI was excellent [40].

Cefixime has been in use for over a decade, with varying degrees of effectiveness all around the world. Globally, pathogens that cause community-acquired illnesses are beginning to show resistance to the first-line prescribed antibiotics. Since antibiotic resistance is increasing, several articles suggest modifying first-line empirical therapy. In certain countries, cefixime is currently the first antibiotic prescribed for the treatment of URTIs, LRTIs, and UTIs [41-46].

Cefixime prescribing pattern

Cefixime is commonly prescribed for various infections, including urinary tract infections (UTIs), upper respiratory tract bacterial infections, *Neisseria gonorrhoeae* genital infections, and typhoid fever [47-50]. The prescribing pattern of cefixime in different infections varies based on the specific infection being treated. A multicenter study conducted in Karachi assessing drug-related problems in patients with stroke revealed that cefixime was prescribed in 47.6% of patients for the treatment of hospital-acquired and stroke-associated infections [51]. In Senegal, cefixime was prescribed to 31.2% of patients with uncomplicated bronchiolitis [52].

A Serbian study on daily antibiotic prescriptions for invasive hospital pathogens revealed that the most commonly prescribed antibiotics included azithromycin (15%), levofloxacin (13%), and cefixime (12%) [53]. Cefixime was one of the most commonly used antibiotics in all regions, particularly in those with higher antibiotic consumption. Evaluation of national averages showed a two-to-three times increase in the usage of cefixime from 2019 to 2021, indicating its significant consumption trend nationally. Cefixime alone constituted one-fourth (25%) of the cumulative consumption of antibiotics in Sindh, while Multan had the lowest cefixime usage compared to other regions in Pakistan [54].

## Conclusions

Cefixime seems to be a potent antibacterial agent that can be suggested as a first-choice medication for the majority of upper respiratory tract infections. Cefixime exhibits good antibacterial activity against pathogens that cause common respiratory tract infections, like acute otitis media, acute sinusitis, and tonsillopharyngitis. Quick treatment of the acute phase of the disease would keep cases from becoming chronic with resistant polymicrobial infections. Clinical research demonstrates that cefixime is at least as efficient as conventional first-line antibiotic therapy for each of these conditions. If broad-ranging antibiotics are misused, as is the case with all antibiotics, resistance to them might increase. Cefixime is a safe option for the empirical treatment of bacterial respiratory tract infections, particularly in those cases where conventional antimicrobial therapy shows signs of resistance.

## References

[1] Pandey N, Cascella M (2023). Beta-Lactam Antibiotics. https://www.ncbi.nlm.nih.gov/books/NBK545311/.

[2] Nakajima S (2003). The origin of cephalosporins (Article in Japanese). Yakushigaku Zasshi.

[3] Jones J (2008). The life and work of Guy Newton (1919-1969). J Pept Sci.

[4] El-Shaboury SR, Saleh GA, Mohamed FA, Rageh AH (2007). Analysis of cephalosporin antibiotics. J Pharm Biomed Anal.

[5] Klein NC, Cunha BA (1995). The selection and use of cephalosporins: a review. Adv Ther.

[6] Sunjaya DB, Lennon RJ, Shah VH, Kamath PS, Simonetto DA (2019). Prevalence and predictors of third-generation cephalosporin resistance in the empirical treatment of spontaneous bacterial peritonitis. Mayo Clin Proc.

[7] García-Rodríguez JA, Muñoz Bellido JL, García Sánchez JE (1995). Oral cephalosporins: current perspectives. Int J Antimicrob Agents.

[8] Tan BJ (1995). Cefixime use in children: when and why. Can J Infect Dis.

[9] Sullins AK, Abdel-Rahman SM (2013). Pharmacokinetics of antibacterial agents in the CSF of children and adolescents. Paediatr Drugs.

[10] Levenstein J, Summerfield PJ, Fourie S, Brink G, Michaelides B, Murray E, Naidoo N (1986). Comparison of cefixime and co-trimoxazole in acute uncomplicated urinary tract infection. A double-blind general practice study. S Afr Med J.

[11] Parmar PM, Solanki VV, Barvaliya MJ, Chavada BC, Tripathi CR (2017). Cephalosporins associated pseudomembraneous colitis in an elderly male patient -w a case report. Curr Drug Saf.

[12] Khan DA, Banerji A, Bernstein JA (2019). Cephalosporin allergy: current understanding and future challenges. J Allergy Clin Immunol Pract.

[13] Grossjohann B, Eichler P, Greinacher A, Santoso S, Kroll H (2004). Ceftriaxone causes drug-induced immune thrombocytopenia and hemolytic anemia: characterization of targets on platelets and red blood cells. Transfusion.

[14] Kim KB, Kim SM, Park W, Kim JS, Kwon SK, Kim HY (2012). Ceftiaxone-induced neurotoxicity: case report, pharmacokinetic considerations, and literature review. J Korean Med Sci.

[15] Abe S (2017). A case of ceftriaxone-associated biliary pseudolithiasis in an elderly patient with renal dysfunction. IDCases.

[16] Harr T, French LE (2010). Toxic epidermal necrolysis and Stevens-Johnson syndrome. Orphanet J Rare Dis.

[17] Kumar V, Kalaiselvan V, Kumar AP, Saurabh A, Thota P, Sidhu S, Medhi B (2018). Cefixime-associated acute generalized exanthematous pustulosis: rare cases in India. Indian J Pharmacol.

[18] Akbayrak A, Yazar C, Alev Deresoy F, Sencan M, Yildiz Seckin H, Kutlu O (2022). Acute localized exanthematous pustulosis because of cefixime in a child: case report and review of pediatric cases. Int J Dermatol.

[19] Jama-Kmiecik A, Frej-Mądrzak M, Sarowska J, Choroszy-Król I (2016). Pathogens causing upper respiratory tract infections in outpatients. Adv Exp Med Biol.

[20] Pettigrew MM, Gent JF, Pyles RB, Miller AL, Nokso-Koivisto J, Chonmaitree T (2011). Viral-bacterial interactions and risk of acute otitis media complicating upper respiratory tract infection. J Clin Microbiol.

[21] Kho BP, Ong CM, Tan FT, Wee CY (2013). Antibiotic prescribing for upper respiratory tract infections in sarawak district hospitals. Med J Malaysia.

[22] Costelloe C, Metcalfe C, Lovering A, Mant D, Hay AD (2010). Effect of antibiotic prescribing in primary care on antimicrobial resistance in individual patients: systematic review and meta-analysis. BMJ.

[23] Don M, Valent F, Korppi M, Canciani M (2009). Differentiation of bacterial and viral community-acquired pneumonia in children. Pediatr Int.

[24] Hendaus MA, Jomha FA, Alhammadi AH (2015). Virus-induced secondary bacterial infection: a concise review. Ther Clin Risk Manag.

[25] Pavia AT (2011). Viral infections of the lower respiratory tract: old viruses, new viruses, and the role of diagnosis. Clin Infect Dis.

[26] Elwell C, Mirrashidi K, Engel J (2016). Chlamydia cell biology and pathogenesis. Nat Rev Microbiol.

[27] Käding N, Szaszák M, Rupp J (2014). Imaging of Chlamydia and host cell metabolism. Future Microbiol.

[28] Pettigrew MM, Gent JF, Revai K, Patel JA, Chonmaitree T (2008). Microbial interactions during upper respiratory tract infections. Emerg Infect Dis.

[29] Ahovuo-Saloranta A, Rautakorpi UM, Borisenko OV, Liira H, Williams JW Jr, Mäkelä M (2014). Antibiotics for acute maxillary sinusitis in adults. Cochrane Database Syst Rev.

[30] Wald ER, Applegate KE, Bordley C (2013). Clinical practice guideline for the diagnosis and management of acute bacterial sinusitis in children aged 1 to 18 years. Pediatrics.

[31] Arumugham VB, Gujarathi R, Cascella M (2023). Third-Generation Cephalosporins. https://www.ncbi.nlm.nih.gov/books/NBK549881/.

[32] Sader HS, Jacobs MR, Fritsche TR (2007). Review of the spectrum and potency of orally administered cephalosporins and amoxicillin/clavulanate. Diagn Microbiol Infect Dis.

[33] Fenoll A, Giménez MJ, Robledo O (2008). In vitro activity of oral cephalosporins against pediatric isolates of Streptococcus pneumoniae non-susceptible to penicillin, amoxicillin or erythromycin. J Chemother.

[34] HaghiAshtiani MT, Sadeghian M, Nikmanesh B (2014). Antimicrobial susceptibility trends among Streptococcus pneumoniae over an 11-year period in an Iranian referral children hospital. Iran J Microbiol.

[35] Fenoll A, Giménez MJ, Robledo O, Aguilar L, Tarragó D, Granizo JJ, Martín-Herrero JE (2008). Influence of penicillin/amoxicillin non-susceptibility on the activity of third-generation cephalosporins against Streptococcus pneumoniae. Eur J Clin Microbiol Infect Dis.

[36] Jansen WT, Verel A, Beitsma M, Verhoef J, Milatovic D (2008). Surveillance study of the susceptibility of Haemophilus influenzae to various antibacterial agents in Europe and Canada. Curr Med Res Opin.

[37] Mandell LA, Wunderink RG, Anzueto A (2007). Infectious Diseases Society of America/American Thoracic Society consensus guidelines on the management of community-acquired pneumonia in adults. Clin Infect Dis.

[38] Lau RC, Doyle JJ, Freedman MH, King SM, Richardson SE (1994). Early discharge of pediatric febrile neutropenic cancer patients by substitution of oral for intravenous antibiotics. Pediatr Hematol Oncol.

[39] Ige OM, Okesola AO (2015). Comparative efficacy and safety of cefixime and ciprofloxacin in the management of adults with community-acquired pneumonia in Ibadan, Nigeria. Ann Ib Postgrad Med.

[40] Dreshaj Sh, Doda-Ejupi T, Tolaj IQ (2011). Clinical role of Cefixime in community-acquired infections. Prilozi.

[41] Ludwig E (1998). Cefixime in the treatment of respiratory and urinary tract infections. Chemotherapy.

[42] Negri MC, Morosini MI, Loza E, Baquero F (2000). Perspectives of oral cephalosporins in upper respiratory tract infections. ClinMicrobiol Infect.

[43] Principi N (2000). Oral cephalosporins in the treatment of acute otitis media. Clin Microbiol Infect.

[44] Garcia Garcia MI, Munoz Bellido JL, Garcia Rodriguez JA (2007). In vitro susceptibility of community-acquired urinary tract pathogens to commonly used antimicrobial agents in Spain: a comparative multicenter study (2002-2004). J Chemother.

[45] Kumamoto Y, Tsukamoto T, Matsukawa M (2005). Comparative studies on activities of antimicrobial agents against causative organisms isolated from patients with urinary tract infections (2003). I. Susceptibility distribution (Article in Japanese). Jpn J Antibiot.

[46] Jacobs MR, Felmingham D, Appelbaum PC, Grüneberg RN (2003). The Alexander Project 1998-2000: susceptibility of pathogens isolated from community-acquired respiratory tract infection to commonly used antimicrobial agents. J Antimicrob Chemother.

[47] Fazaludeen Koya S, Hasan Farooqui H, Mehta A, Selvaraj S, Galea S (2022). Quantifying antibiotic use in typhoid fever in India: a cross-sectional analysis of private sector medical audit data, 2013-2015. BMJ Open.

[48] Mehta SN, Stafylis C, Tellalian DM (2020). Clinical trial protocol to evaluate the efficacy of cefixime in the treatment of early syphilis. Trials.

[49] Naik B, Bhagyashree B, Bhagyashree S (2023). A study on bacterial etiology and antibiotic utilization pattern among inpatient with urinary tract infections. Ind J Pharm Pract.

[50] Kelly J, Toy T, Dersch-Mills D, Stang AS, Constantinescu C, Robinson JL (2023). Antibiotic prescribing practices for urinary tract infection in a pediatric emergency department: is this a problem worth Cefix-ing?. Can J Hosp Pharm.

[51] Ali M, Shoaib MH, Nesar S (2024). A prospective observational study of estimating drug related problems and clinical outcomes in subtypes of stroke patients. PLoS One.

[52] Ardillon A, Ramblière L, Kermorvant-Duchemin E (2023). Inappropriate antibiotic prescribing and its determinants among outpatient children in 3 low- and middle-income countries: a multicentric community-based cohort study. PLoS Med.

[53] Medic D, Bozic Cvijan B, Bajcetic M (2023). Impact of antibiotic consumption on antimicrobial resistance to invasive hospital pathogens. Antibiotics (Basel).

[54] Mustafa T, Niazi MR, Lakdawala Z, Mirza S (2023). Regional and national trends in consumption of antimicrobials in Pakistan; pre and post-COVID (2019-2021). Clin Infect Dis.

